# Escargot controls the sequential specification of two tracheal tip cell types by suppressing FGF signaling in *Drosophila*

**DOI:** 10.1242/dev.133322

**Published:** 2016-11-15

**Authors:** Guangxia Miao, Shigeo Hayashi

**Affiliations:** 1 Laboratory for Morphogenetic Signaling, RIKEN Center for Developmental Biology, 2-2-3 Minatojima-minamimachi, Chuo-ku, Kobe, Hyogo 650-0047, Japan; 2 Department of Biology, Kobe University Graduate School of Science, 1-1 Rokkodai-cho, Nada-ku, Kobe, Hyogo 657-8051, Japan

**Keywords:** Tubulogenesis, Tip cell, Cell migration, FGF signaling, ERK signaling

## Abstract

Extrinsic branching factors promote the elongation and migration of tubular organs. In the *Drosophila* tracheal system, Branchless (*Drosophila* FGF) stimulates the branching program by specifying tip cells that acquire motility and lead branch migration to a specific destination. Tip cells have two alternative cell fates: the terminal cell (TC), which produces long cytoplasmic extensions with intracellular lumen, and the fusion cell (FC), which mediates branch connections to form tubular networks. How Branchless controls this specification of cells with distinct shapes and behaviors is unknown. Here we report that this cell type diversification involves the modulation of FGF signaling by the zinc-finger protein Escargot (Esg), which is expressed in the FC and is essential for its specification. The dorsal branch begins elongation with a pair of tip cells with high FGF signaling. When the branch tip reaches its final destination, one of the tip cells becomes an FC and expresses Esg. FCs and TCs differ in their response to FGF: TCs are attracted by FGF, whereas FCs are repelled. Esg suppresses ERK signaling in FCs to control this differential migratory behavior.

## Abstract (precis)

**Summary:** The migratory behavior of tracheal fusion cells is controlled by the FGF-induced expression of the transcription factor Escargot, which subsequently suppresses ERK signaling.

## INTRODUCTION

Tubular organs allow for circulation through blood vessels and promote the exchange of gases in the mammalian lung and insect trachea, thereby increasing the oxygen uptake efficiency in large animals that have a high volume to surface ratio and are otherwise incapable of effective respiration. The development of tubular organs is triggered by the expression of secreted signaling molecules. The expression pattern of these molecules, either singly or in combination, acts as a branching signal by instructing signal-receiving tubule cells to extend and migrate toward a specific destination. In angiogenesis the major branching signal is Vascular endothelial growth factor (VEGF); in the respiratory system, Fibroblast growth factor (FGF) signaling is crucial in both mammalian lung and insect trachea development (Cardoso and Lü, 2006; Hoeben et al., 2004; Metzger and Krasnow, 1999). In each case, branching signals activate a receptor tyrosine kinase, which transduces the Ras-ERK signaling cassette and promotes cell proliferation and cell motility (Koch and Claesson-Welsh, 2012; Metzger and Krasnow, 1999; Morrisey and Hogan, 2010). FGF signaling is also crucial for guiding cell migration during vertebrate and invertebrate gastrulation. FGF4 and FGF8 in the early mouse and chick gastrula direct the migration of epiblast cells out of the primitive streak (Ciruna and Rossant, 2001; Yang et al., 2002).

In angiogenesis, branching signals immediately induce the specification of endothelial tip cells that lead the sprouting and migration of new vessel branches (Phng and Gerhardt, 2009). Tip cells, which are located at the leading edge of extending sprouts, are characterized by extensive filopodia, by their migratory activity, and by the expression of Dll4, the transmembrane ligand for Notch signaling. The activation of cells adjacent to the tip cells activates Notch signaling; these cells adopt the fate of stalk cells (SCs), which have fewer filopodia, are less motile, and follow the tip cells in the sprouting process (Hellström et al., 2007). Through a lateral inhibition mechanism, each sprout is led by a single tip cell that is followed by SCs.

Branching morphogenesis of the *Drosophila* trachea system is governed by FGF signaling (Ghabrial et al., 2003; Sutherland et al., 1996). Tracheal primordia are specified in each side of the T2 to A8 segments as a cluster of 60-80 cells. After invagination, the tracheal primordial cells start expressing the FGF receptor (FGFR) Breathless (Btl) (Klambt et al., 1992). Branchless (Bnl; *Drosophila* FGF), which is expressed at specific locations of the mesodermal and ectodermal tissues surrounding each tracheal primordium, activates FGF signaling in a subset of tracheal cells that form the primary branches (Klambt et al., 1992; Sutherland et al., 1996). Delta and an active phosphorylated form of ERK (dpERK) are strongly expressed at the tip of each primary branch (Gabay et al., 1997; Ikeya and Hayashi, 1999). Through lateral inhibition, Delta-positive cells converge into a single cell in each branch, and this cell has numerous filopodia and strong migratory activity (Klambt et al., 1992; Llimargas, 1999).

Two types of cells differentiate from the tip of migrating tracheal branches at later embryonic stages. Fusion cells (FCs) form anastomoses in the dorsal trunk, lateral trunk, dorsal branch, cephalic branch and ventral branch by adhering in a pairwise manner and converting into a torus shape to connect the lumen (Caviglia and Luschnig, 2014; Gervais et al., 2012; Samakovlis et al., 1996b; Tanaka-Matakatsu et al., 1996). Terminal cells (TCs) differentiate to extend long cytoplasmic extensions (terminal branches) that cover target tissues and exchange air with the intracellular lumen (Guillemin et al., 1996; Samakovlis et al., 1996a). After the primary branches are specified and have navigated toward their specific destinations, FGF signaling performs a second tracheal function, that of promoting TC differentiation (Gervais and Casanova, 2011; Lee et al., 1996; Reichman-Fried and Shilo, 1995) and navigation (Miao and Hayashi, 2015). Some tracheal branches develop both an FC and a TC and extend the terminal branch from the tube connection point. Although some mechanisms that suppress the emergence of tip cells have been elucidated (Caviglia and Luschnig, 2013; Chen et al., 1998), how the two types of tip cells are selected from the pool of FGF-activated, migration-competent branch tip cells after Notch-induced lateral inhibition is not understood.

Here we addressed the cell type diversification of FGF-activated branch tip cells. We show that the early FC marker Escargot (Esg) plays a central role in tip cell diversification by promoting the expression of another FC gene, *dysfusion* (*dys*; *dysf* – FlyBase), and suppressing expression of the TC gene *Drosophila serum response factor* (*DSRF*; *blistered* – FlyBase). In addition, Esg suppresses FGF signaling partly by downregulating the FGF signal transducer Downstream of FGF (Dof; Stumps – FlyBase). Therefore, the fusion competence of specific tracheal branches is acquired through suppression of the default TC fate by Esg.

## RESULTS

### Dorsal branch development in *Drosophila*

**Fig. 1.**
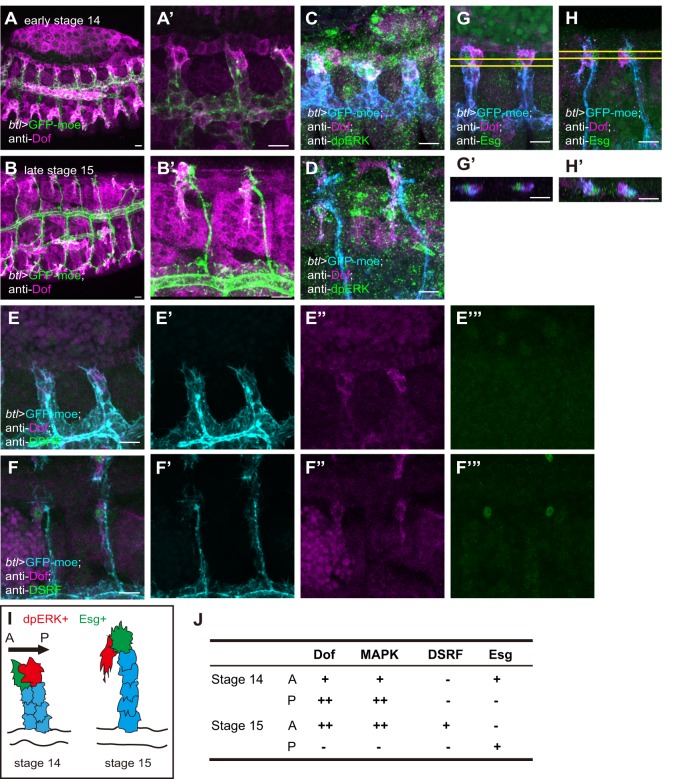
**Normal dorsal branch (DB) development in *Drosophila* embryos.** Anti-Dof staining (A-B′) and co-staining for Dof and dpERK (C,D), DSRF (E-F‴) or Esg (G-H′) of *btl-Gal4*-driven *UAS-GFP-moe Drosophila* embryos. (A,C,E,G) Early stage 14. (B,D,F,H) Late stage 15. (A′,B′) Magnified views of A,B. (E′-E‴,F′-F‴) Single-channel images of E,F. (G′,H′) *x-z* views of the area between the yellow lines in G,H. Scale bars: 10 µm. (I) Schematic of the DB at stages 14 and 15, showing Esg-positive cells (green), dpERK-positive cells (red) and stalk cells (SCs; blue). (J) Summary of gene expression patterns in DB tip cells. A, anterior; P, posterior.

To investigate the mechanism of divergent cell fate determination under FGF signaling, we focused on the dorsal branch (DB), which migrates dorsally and fuses with another DB from the contralateral side at the dorsal midline (Kato et al., 2004; Samakovlis et al., 1996b). At stage 15, the DB tips have reached the dorsal margin of the dorsal epidermis (DE) and remain fixed to the same location in the DE (Kato et al., 2004). DB tips are brought to the dorsal midline by the dorsal closure movement (Kato et al., 2004, 2016). At this stage, three cell types are distinguished by their specific shapes and marker gene expression (Fig. 1I): the FC (green), which is located in the dorsalmost position and mediates branch fusion; the TC (red), which is located on the anterior side of the DB tip and sprouts a long terminal branch ventrally along the compartment boundary; and the SCs (blue), which are tandemly aligned behind the FC (Fig. 1I). The FC and TC are marked by expression of the transcription factors Esg and DSRF, respectively (Fig. 1F,H,J) (Guillemin et al., 1996; Tanaka-Matakatsu et al., 1996). At early stage 14, the DB tip consists of a pair of cells, the anteriormost of which expresses Esg (Fig. 1G,I,J) but not DSRF at any detectable level (Fig. 1E-E‴).

To elucidate the role of FGF signaling in DB development, we examined the expression of Dof (Imam et al., 1999; Michelson et al., 1998; Vincent et al., 1998), an intracellular protein that acts downstream of Btl (FGFR) and upstream of Ras. Dof is specifically expressed in cells expressing either Btl or Heartless (Htl), and is needed for the activation of MAPK signaling via FGF signaling (Imam et al., 1999; Michelson et al., 1998; Vincent et al., 1998). Dof is first expressed in all tracheal cells during stages 10 to 12, and is then strongly expressed in the tip cells at stage 13 (Fig. S1). Dof expression in the trachea is similar to that of *dof* and *btl* RNA (Ohshiro and Saigo, 1997; Vincent et al., 1998). At stage 14, Dof expression was concentrated in the TC, although it was still expressed in the FC and, though weakly, also in SCs (Fig. 1A,A′). After stage 15, when the TC extends long terminal branches, Dof was expressed only in the TC (Fig. 1B,B′), which also activated ERK (Fig. 1C,D). These observations suggested that Dof can be used as a marker to trace FGF signaling in tracheal cells.

### Differential roles of Esg and Dys in FC specification

**Fig. 2.**
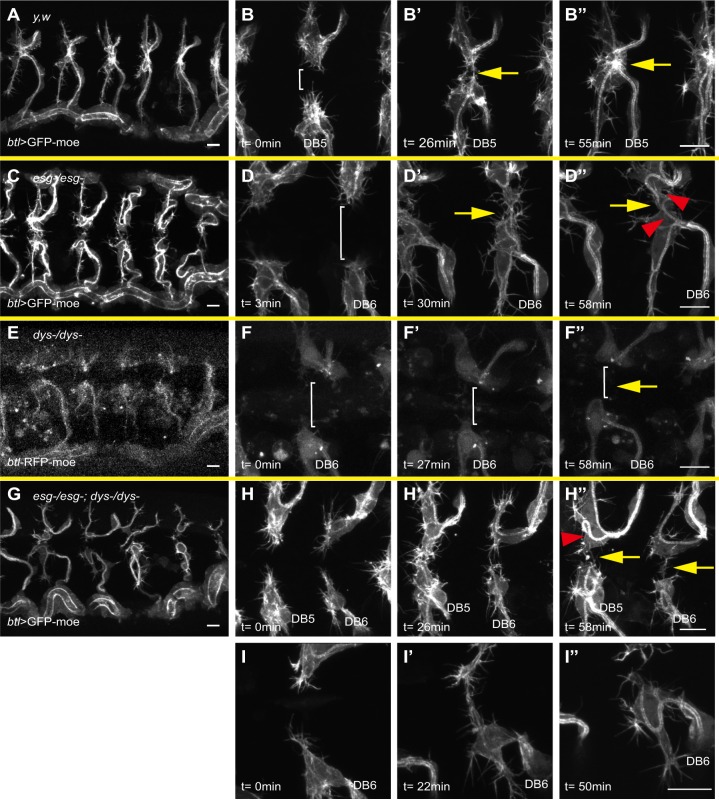
**Differential roles of Esg and Dys in fusion cell (FC) specification.** Overall views of a *btl>GFP-moe* embryo (A) and an *esg* mutant (C), *dys* mutant (E), and *esg*;*dys* double mutant (G). High-magnification views of *btl>GFP-moe* (B-B″), *esg* mutant (D-D″), *dys* mutant (F-F″), and *esg*;*dys* double-mutant (H-I″) are shown. Note that *btl-RFP-moe* was used as a marker for the *dys* mutant, whereas the others are marked with *btl>GFP-moe*. Brackets indicate the gap between the FC and its partner cell. Yellow arrows indicate prospective fusion points. Red arrowheads indicate ectopic terminal cell (TC)-like cells. Time (*t*) is relative to the start of live imaging. Scale bars: 10 µm.

Our data indicated that the onset of Esg expression coincides with FC specification. Dys is a basic helix-loop-helix (bHLH)-PAS transcription factor that is expressed in FCs (Jiang and Crews, 2003, 2006). Although both Esg and Dys are required for DB fusion, whether their roles in the fusion process overlap has been not addressed. We used live imaging to compare the phenotypes of *esg* and *dys* mutant embryos. In control embryos at late stage 15, FCs from each side extend numerous filopodia and contact each other at the dorsal midline, establish new cell adhesion interfaces, and change into a compact torus shape with a very short lumen to connect the two DBs (Fig. 2A-B″, Movie 1) (Gervais et al., 2012; Samakovlis et al., 1996b; Tanaka-Matakatsu et al., 1996). In *esg* mutant embryos, FCs reached the dorsal midline and contacted each other but failed to establish new adhesion interfaces; they instead continued to elongate into an extensively winding form with what appeared to be a TC-like internal lumen (Fig. 2C-D″, Movie 2). This live imaging analysis confirmed a previous observation, based on fixed preparations, that *esg* mutant FCs acquire a TC-like character (Samakovlis et al., 1996b; Tanaka-Matakatsu et al., 1996). In *dys* mutant embryos, the FCs also failed to complete fusion (Jiang and Crews, 2006).

High-resolution live imaging revealed differences in the FC phenotypes between the *dys* and *esg* mutants. The FCs in *dys* mutants, as visualized with *btl-RFP-moe*, formed fewer filopodia than controls and *esg* mutants labeled with the same marker, and migration of FCs in the *dys* mutant was retarded: in 54% of the metameres (22 out of 41 fusion points, 11 embryos) the FCs failed to reach the dorsal midline or to contact other FCs [Fig. 2E-F″, Fig. S2, Movie 3; contact failure rate in control was 0.02% (1/59 fusion points, 11 embryos), whereas in *esg* mutants it was 12.5% (2/16 fusion points, 3 embryos)]. No TC-like behavior or luminal structures were observed in *dys* mutant FCs (Fig. 2F″). These observations indicate that *esg* and *dys* play distinct roles in tracheal branch fusion.

### Epistatic relationship of *esg*, *dys* and *DSRF*

**Fig. 3.**
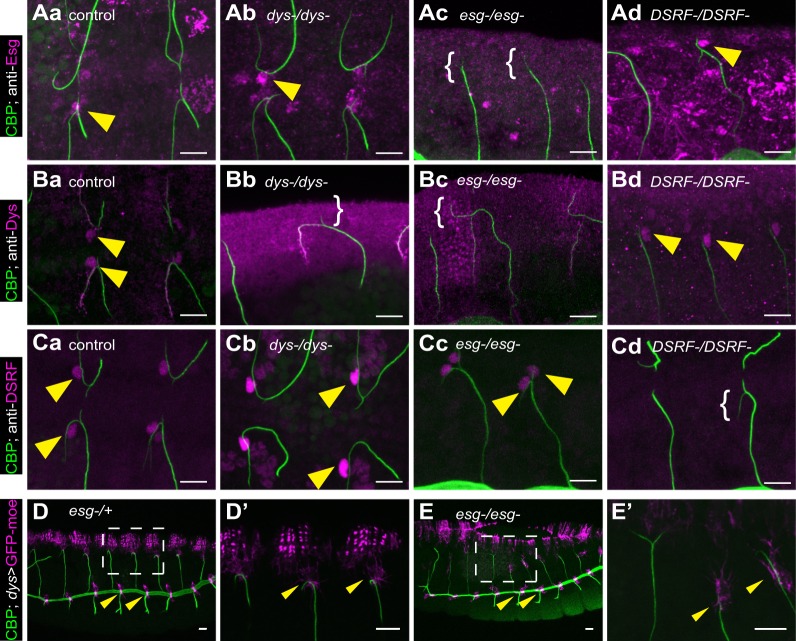
**Epistatic relationship of *esg*, *dys* and *DSRF*.** Immunostaining with anti-Esg (Aa-d), anti-Dys (Ba-d) or anti-DSRF (Ca-d) in control (Aa,Ba,Ca), *dys* mutant (Ab,Bb,Cb), *esg* mutant (Ac,Bc,Cc) and *DSRF* mutant (Ad,Bd,Cd) embryos. The expression pattern of the *dys* enhancer-Gal4 construct was detected with UAS-GFP-moe reporter in *esg* mutant heterozygotes (D,D′) and homozygotes (E,E′). The boxed region in D,E is magnified in D′,E′ to show the DB. Arrowheads indicate Esg, Dys, DSRF or GFP. The anti-Dys antibody gave non-specific labeling of the terminal branch that persisted in *dys* mutant embryos. Brackets indicate the absence of Esg, Dys or DSRF expression. Chitin is in green (CBP). Scale bars: 10 µm.

To understand the relationship between *esg* and *dys*, it was important to clarify their epistatic relationship. Esg expression in DB FCs was unaltered in the *dys* mutant background (Fig. 3Aa,b), consistent with a previous report (Jiang and Crews, 2003) that an *esg-lacZ* reporter remains expressed in embryos injected with double-stranded *dys* transcripts. Jiang and Crews (2003) reported that Dys expression is lost in FCs of the DB, the lateral trunk, and the first three ganglionic branches in *esg* mutant embryos, but is maintained in the dorsal trunk (DT). Downregulation of Dys expression in the FC of DBs was confirmed (Fig. 3Bc). However, the immunostaining experiment was insufficient to verify whether Dys expression was totally lost in *esg* mutants. We therefore used the more sensitive method of monitoring the activity of the *dys* FC-specific enhancer (Jiang et al., 2010; Jenett et al., 2012), which we found remained active in DB and DT of *esg* mutant trachea (Fig. 3D,E). We concluded that *esg*-dependent and *esg*-independent pathways coordinately regulate the FC-specific expression of *dys*.

**Fig. 4.**
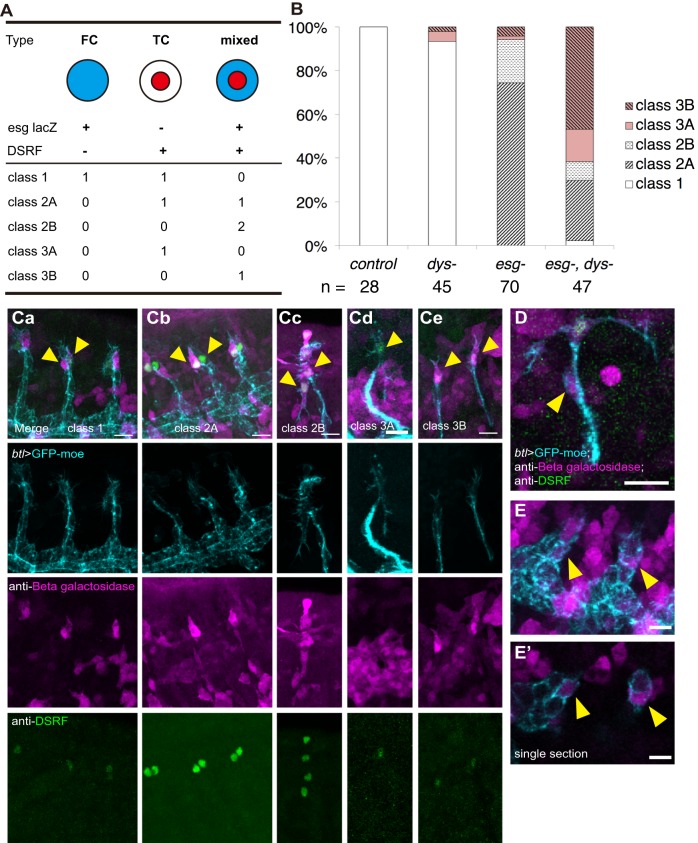
**Synergistic interaction of *esg* and *dys* mutants.** (A) Five classes of tip cells and their gene expression patterns in the different mutants. (B) The frequency of the tip cell phenotypes in mutants. (Ca-e) Anti-β-gal (*esg*-*lacZ*) and anti-DSRF staining for the five classes. (D-E′) *esg-lacZ* expression in SCs in *esg*;*dys* double-mutant embryos. (D) Stage 16. (E) Stage 14. (E′) Single section showing the localization of *lacZ*-positive cells. Arrowheads indicate tip cells (Ca-e) or *lacZ*-positive SCs (D-E′). Scale bars: 10 µm.

We next studied the expression of the TC marker DSRF. In *esg* mutants, DSRF-positive cells were duplicated in 94% of the DBs (66 out of 70 DBs, 10 embryos; Fig. 3Cc, Fig. 4B), consistent with a model in which Esg negatively regulates *DSRF* and suppresses the TC phenotype in FCs (Samakovlis et al., 1996b). By contrast, the number of DSRF-positive cells was unchanged in *dys* mutants (Fig 3Cb, Fig 4B). Esg and Dys expression was unaltered in *DSRF* mutants (Fig. 3Ad,Bd), suggesting that Esg regulates *dys* and *DSRF* independently.

To further characterize the changes in tip cell specification in mutant embryos, we studied *lacZ* expression under the control of the *esg* enhancer (*esg-lacZ*). The long persistence of the *lacZ* product β-gal permits monitoring of the current and past transcriptional states of *esg* in mutant cells. In control embryos, the DB tip contained pairs of DSRF^+^ cells (TC type) and *esg-lacZ^+^* single-positive cells (FC type) (class 1, Fig. 4A,B,Ca). In 74% of the *esg* mutant DBs, TC type cells were paired with DSRF and *esg-lacZ* double-positive cells (mixed identity type, Fig. 4A,B,Cb; 52 out of 70 DBs, 10 embryos); we called this combination class 2A. This finding implied that one of the tip cells, once the *esg* enhancer was activated, expressed DSRF. In 20% of the cases, both tip cells were of the mixed type (class 2B, Fig. 4A,B,Cc; 14 out of 70 DBs, 10 embryos). In *dys* embryos, the majority of the DB tip cells (93%) were class 1, with a minor fraction of other classes (42 out of 45 DBs, 7 embryos; Fig. 4B).

To further verify this epistasis model, we generated *esg*;*dys* double mutants. The double-mutant trachea showed a variety of defects, including the appearance of intracellular lumen in the leading cells (similar to the *esg* mutant) and delayed DB migration (similar to the *dys* mutant) (Fig. 2G,H″, Movie 4). Class 2 tip cell phenotypes were seen in 36% of the double-mutant DBs (Fig. 4B; 17 out of 47 DBs, 5 embryos). In addition, we observed a novel phenotype in which the FCs were lost (class 3A and 3B, single tip cells of TC or mixed type; Fig. 4Cd,e). The remaining TCs sometimes bifurcated and extended dorsal and ventral protrusions (Fig. 2G,I-I″, Movie 5). The single tip cell in this phenotype was characterized by DSRF expression, and was seen in 62% of the double-mutant DBs (class 3A and 3B, 29 out of 47 DBs, 5 embryos; Fig. 4B,Cd,e). In some double-mutant DBs, *esg-lacZ*^+^ cells were found in the DB stalk (Fig. 4D-E′; 3 out of 29 single tip cell DBs), indicating that misplacement of prospective FCs in the stalk accounts for some cases of the single tip cell phenotype.

Based on these observations, we concluded that *esg* and *dys* have distinct roles in FC specification: *esg* specifies fusion competence by promoting E-cadherin expression (Tanaka-Matakatsu et al., 1996) and suppressing TC differentiation (Samakovlis et al., 1996b), whereas *dys* specifies migratory competence by promoting filopodia formation (Jiang and Crews, 2006). The simultaneous loss of *esg* and *dys* caused FC mis-specification, resulting in transformation of the cells into TCs (class 2, 36%) or a loss of mutant FCs (class 3, 62%). In either case, the DB dorsal extension was delayed.

### Esg suppresses FGF signaling in tracheal tip cells

**Fig. 5.**
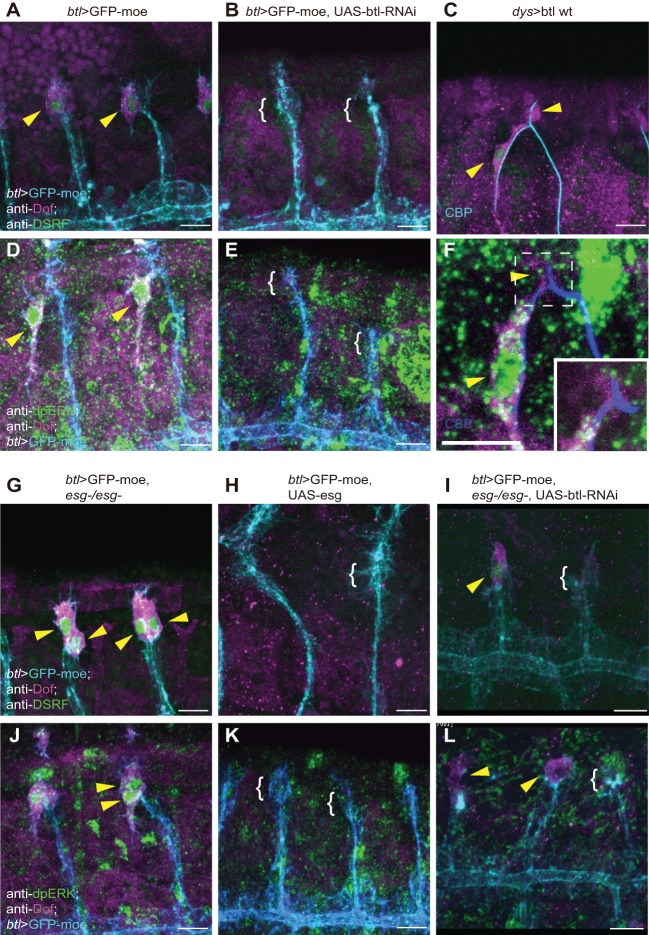
**Esg suppresses FGF signaling in tracheal tip cells.** Immunostaining with anti-Dof plus anti-DSRF (A-C,G-I) or plus anti-dpERK (D-F,J-L) of (A,D) *btl>GFP-moe*, (B,E) *btl>GFP-moe, UAS-btl-RNAi*, (C,F) *dys>btl wt*, (G,J) *btl>GFP-moe, esg^−/−^*, (H,K) *btl>GFP-moe, UAS-esg* and (I,L) *btl>GFP-moe, UAS-btl-RNAi*, *esg^−/−^* embryos. The boxed region in F is magnified in the inset to show a single section of a DB tip. Arrowheads indicate Dof, DSRF or dpERK. Brackets show the absence of Dof, DSRF or dpERK expression. Scale bars: 10 µm.

As shown above, Esg, but not Dys, suppresses DSRF expression in FCs (Fig. 3Cb,c). Since DSRF expression depends on FGF (Bnl) signaling (Sutherland et al., 1996), we examined whether Esg regulates FGF signaling. We first characterized the regulation of FGF signaling in FCs by altering the expression of Btl (FGFR). When we reduced Btl expression using RNAi constructs driven by *btl-Gal4*, we found that the FGF signaling markers Dof, dpERK and DSRF were strongly inhibited (Fig. 5B,E, compare with Fig. 5A,D). Remarkably, Btl overexpression caused by an FC-specific *dys-Gal4* increased Dof expression in FCs. This treatment, however, did not induce ectopic ERK activity or DSRF expression (Fig. 5C,F). The failure of ectopic Btl to increase FGF signaling might be due to the limited source of Bnl.

Next, we studied the effect of Esg on Dof, dpERK and DSRF. In *esg* mutants, Dof and dpERK were ectopically elevated in FCs (Fig. 5G,J). This elevation of Dof and dpERK was due to overactivation of FGFR, since ectopic Dof and dpERK were partially reduced by simultaneous downregulation of *btl* (Fig. 5I,L). By contrast, Esg overexpression strongly reduced Dof, dpERK and DSRF in TCs (Fig. 5H,K). These results demonstrated that Esg suppresses FGF signaling at the level of FGFR activation in tracheal tip cells.

### The TC posterior-to-anterior position shift depends on *esg*

We noted that in control stage 14 DBs, when Esg expression was first detected, cells with high ERK activation and Dof expression were positioned posterior to the Esg^+^ tip cells (Fig. 1C,G,G′,I). Later, at stage 15, dpERK^+^ and Dof^+^ cells began expressing DSRF and were found anterior to Esg^+^ cells (Fig. 1D,H,H′,I). To clarify the reason for this positional shift, we monitored FC location relative to other tracheal cells. At the onset of expression of the *esg* enhancer-Gal4 at early stage 14, the FCs were found in direct contact with the epidermis, and prospective TCs were positioned on top of the FCs (Fig. S3A,A″, Movie 6). At late stage 14, prospective TCs moved anteriorly and made direct contact with the epidermis (Fig. S3B-B″). Based on these observations, we concluded that this shift in position involves the migration of prospective TCs with high FGF signaling activity toward the anterior.

We next examined the role of *esg* in TC anterior migration. We counted cells expressing *esg-lacZ* and DSRF, and detected this TC positional shift in 85% of the control embryo DBs (24 out of 28) at stage 15. The TC position shifts occurred at a similarly high frequency in *dys* mutants (90%, 38 out of 42). We also counted the frequency of TC positional shifts in type 2A branches of *esg* mutants. We found that *esg-lacZ^−^* DSRF^+^ cells (authentic TCs) were positioned anterior to *esg-lacZ*^+^ DSRF^+^ cells (prospective FCs transformed into TCs) in 56% of cases (29 out of 52). Therefore, the frequency of anterior positional shifts of prospective TCs was reduced to a near-random level. This suggested that the proper anterior-posterior (AP) positioning of TCs and FCs is established by TC migration that depends on *esg*.

### Esg modulates Bnl-induced cell migration

TCs and FCs had contrasting migratory behaviors at stage 15. While TCs migrated from the posterior to the anterior of the DB tip and extended the terminal branch ventrally, FCs remained near the leading edge of the DE, became polarized dorsally, and extended filopodia to contact FCs coming from the contralateral side. The direction of terminal branch migration is controlled by a combination of Hedgehog and Dpp signaling (Kato et al., 2004). In addition, localized Bnl is an attractive cue for terminal branch migration (Miao and Hayashi, 2015). Since paired TCs and FCs are exposed to very similar levels of signaling ligands, their distinct migratory behaviors must reflect their cell type-specific interpretation of the signaling environment. To test this hypothesis, we compared the FC and TC responses to ectopic Bnl. We previously showed that TCs respond positively, extending the terminal branch toward cells that ectopically express Bnl from a heat-shock (HS) promoter construct induced by laser (Miao and Hayashi, 2015).

**Fig. 6.**
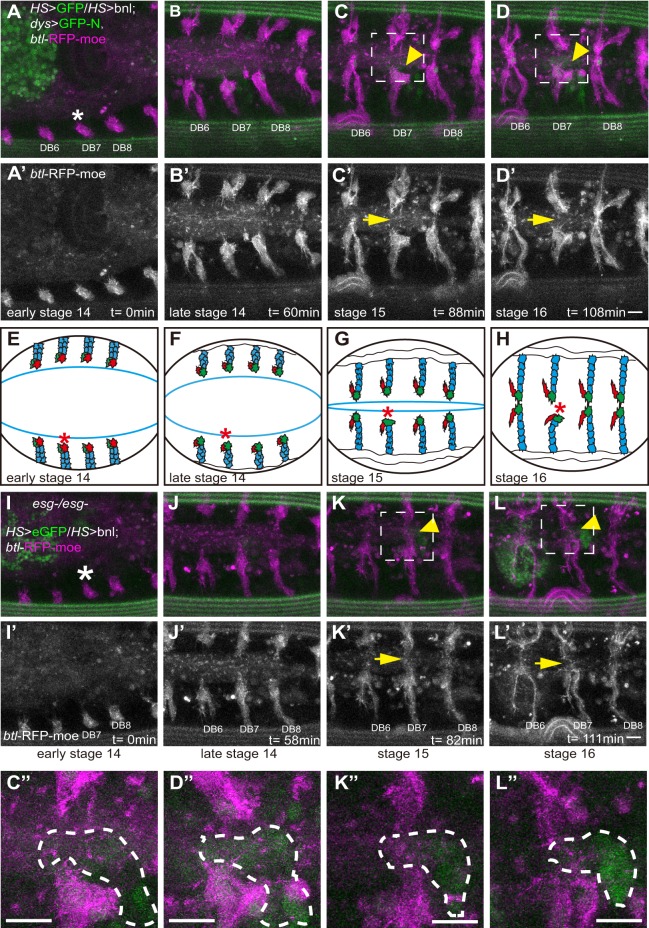
**Esg modulates Bnl-induced cell migration.** (A-D″) Images of *HS-eGFP*, *HS-bnl*, *dys>GFP-N* and *btl-RFP-moe* embryos showing that the induction of ectopic Bnl inhibits the DB fusion process. (I-L′) Ectopic Bnl was induced in the fusion position in an *esg* mutant embryo. (A,A′,I,I′) Early stage 14 embryo. The strong green fluorescence is autofluorescence in yolk cells. (B,B′,J,J′) Late stage 14 embryo. (C,C′,K,K′) Stage 15 embryo. (D,D′,L,L′) Stage 16 embryo. (C″,D″,K″,L″) Magnified views of boxed regions in C,D,K,L. The eGFP-positive cell is outlined (dashed line). Arrowheads indicate HS-eGFP. Arrows indicate prospective fusion points. (E-H) Schematics of the DB fusion process, showing FCs (green), TCs (red) and SCs (blue). Asterisks indicate the heat-shock position. The blue line shows the leading edge. Scale bars: 10 µm.

We used IR-LEGO (Kamei et al., 2009) to induce Bnl ectopically in a prospective fusion position of early stage 14 embryos, and tracked the FC responses by live imaging (Fig. 6A-D′, Movie 7). At stage 14, all of the FCs migrated normally (Fig. 6B,B′). At late stage 15, when the FCs in control branches migrated to the dorsal midline and fused, the migration of the FC adjacent to the ectopic Bnl was arrested (7 out of 10 cases; Fig. 6C-D″) or made a detour around the Bnl-expressing cells (Fig. S5). By contrast, HS-eGFP expression in the prospective fusion position did not affect FC migration and fusion (8 out of 8 cases; Fig. S4, Movie 9). This suggested that FGF signaling inhibits FC migration, in contrast to its role as an attractant for TCs.

To investigate whether *esg* contributes to the inhibitory migratory response to Bnl, we repeated the experiment in *esg* mutant embryos. DB migration was indistinguishable in laser-treated and control segments (Fig. 6I-L′, Movie 8). The prospective FC extended a long terminal branch across the site of ectopic Bnl expression and reached the dorsal midline (5 out of 5 cases; Fig. 6K-L″). These findings indicated that Esg regulates the inhibitory migratory response of FCs to Bnl.

## DISCUSSION

### Esg coordinates the sequential specification of FCs and TCs

**Fig. 7.**
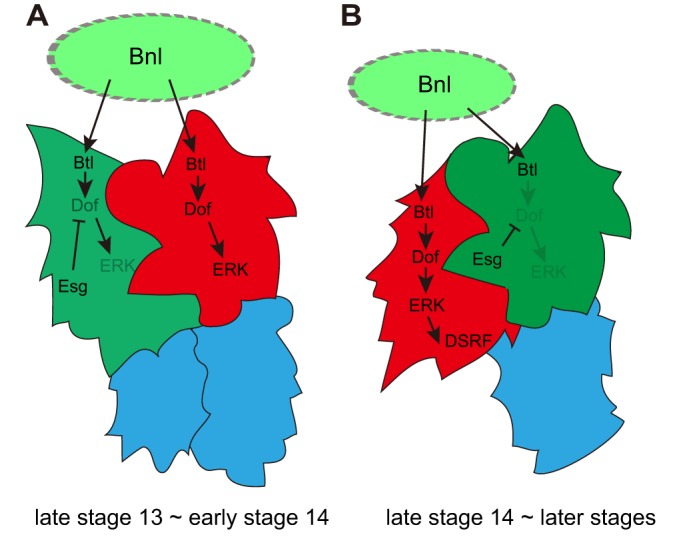
**Esg coordinates the sequential specification of the FC and TC.** Schematics of the regulatory network under FGF (Bnl) signaling and Esg in the DB at late stage 13 to early stage 14 (A) and late stage 14 to subsequent stages (B), showing Esg^+^ cells (green), dpERK^+^ cells (red) and SCs (blue).

In the *Drosophila* tracheal system, the combined actions of Bnl and Wingless specify tip cell fate by stimulating ERK signaling and the expression of *esg* and the Notch ligand *Delta* (Chihara and Hayashi, 2000; Ikeya and Hayashi, 1999; Llimargas, 1999, 2000). Delta is broadly expressed in all tracheal cells and is upregulated in the tip region. Through lateral inhibition, the tip cell fate is restricted to single cells that begin expressing *esg* at stage 13 (Ikeya and Hayashi, 1999; Llimargas, 2000; Steneberg et al., 1999). The rest of the tracheal cells activate Notch signaling and acquire the SC fate by suppressing FC marker genes and ERK signaling (Ikeya and Hayashi, 1999; Llimargas, 1999). After FC specification, the second mode of FGF (Bnl) signaling begins. Btl (FGFR) activation after this stage results in the specification of a TC expressing DSRF (Lee et al., 1996; Sutherland et al., 1996). Our findings show that the Esg-dependent suppression of FGF signaling makes the FC insensitive to Bnl, after which the adjacent tip cell increases its FGF signaling to the highest level found among DB cells and begins specification of the TC fate (Fig. 7A). At late stage 14, prospective TCs shift position to the anterior side and begin expression of the TC marker DSRF (Fig. 7B). Therefore, FCs and TCs are sequentially specified by Bnl, and Esg plays a central role in changing the target of FGF signaling from FCs to TCs.

### Distinct roles of Esg and Dys

Our genetic epistasis analysis demonstrated that *esg*;*dys* double mutants display phenotypes that are more severe than those of the respective single mutants, indicating that *esg* and *dys* have unique functions. High Dys expression in DB FCs depends on *esg* (Jiang and Crews, 2003), but an *esg*-independent pathway through the *dys* FC enhancer stimulates low-level *dys* transcription in an *esg* mutant background. These pathways, together with *dys*-independent *esg* function, collectively specify the FC character. Since DSRF expression was unaltered in *dys* mutants, the suppression of FGF signaling was mainly ascribed to the *dys*-independent, *esg-*dependent pathway. *dys* plays a role in branch migration through stimulating filopodia formation (Jiang and Crews, 2006). Since *esg* lies upstream of *dys* expression, *esg* is placed as a primary determinant of FC fate. It should be noted that *esg* and *dys* functions are dispensable for the fusion of DT, where guidance of FGF-producing cells plays a major role (Wolf et al., 2002).

### Differential control of tip cell migration

We found that the differential migratory behavior of DB tracheal tip cells occurs in two steps. The first is an anteroposterior positional shift in TCs and FCs that occurs at late stage 14 (Fig. 7). The second is a polarized cell protrusion extending along the dorsoventral axis at stage 15, when the FC sends filopodia toward the dorsal midline, and the TC sends the terminal branch ventrally. These differential migratory behaviors can be explained by the sequential action of FGF signaling as follows: at stage 14, *bnl* mRNA is expressed in stripes in each epidermal segment, and the DB tips are associated with the basal surface of *bnl-*positive epidermal cells (Kato et al., 2004). FC differentiation begins just after Esg is expressed in the anterior cell of the tip cell pair (Fig. 7A). FGF signaling in FCs declines as Esg expression increases, and the posterior cell of the tip cell pair, which is the prospective TC, elevates its FGF signaling to the highest level (Fig. 7A) and, attracted to the source of Bnl, migrates over the FC to complete the AP positional change (Fig. 7B).

The second phase of migration starts at stage 15. DSRF expression starts in the TC, which at this time has started to send a terminal branch along the AP compartment boundary. The ventral orientation of the terminal branch is determined by the positioning of the TC body relative to the epidermal region with the highest Bnl expression, the restriction of terminal branch elongation to the Hedgehog-expressing P compartment, and the repulsive effect of Dpp expressed in the dorsal midline (Kato et al., 2004). FCs, by contrast, have low ERK activity and are not positionally restricted by the Bnl source. In this regard, it is surprising that FCs were repulsed by ectopic Bnl induced by a local laser heat shock. In *esg* mutants, the TC-like tip cells transformed from FCs were not repulsed by Bnl. We speculate that, in the presence of Esg, the FGFR ERK signaling branch is suppressed in FCs, while other branches such as PI3K-Akt might still be active and able to instruct a repulsive response to ectopic Bnl. In this regard, Akt phosphorylates Trachealess and regulates its nuclear localization (Jin et al., 2001). However, the role of Akt in tracheal branch migration is not known.

### Esg regulates Dof and FGF signaling

We showed that the expression of Dof protein is elevated in the tip region of tracheal branches. This pattern is similar to that of *dof* and *btl* mRNA and requires Btl (Fig. 1), indicating that Dof expression can be used as a readout of FGFR (Btl) signaling in tracheal cells. Interestingly, Btl overexpression elevated the Dof levels in FCs. Since ERK signaling was not elevated in this condition (probably because the amount of Bnl available for receptor activation, or of other molecules in the signaling cascade, was limited), it is likely that the increased level of Dof observed in the cytoplasm was due to protein stabilization by its interaction with Btl, as occurs in yeast cells (Battersby et al., 2003). We have shown that Esg inhibits a high accumulation of Dof in FCs (Fig. 5). Since the *esg* and *btl* double knockout eliminated ectopic Dof and ERK activation in FCs, hyperactivation of FGFR is likely to be the cause of the *esg* mutant phenotype. Future work should be directed towards elucidating the molecular mechanism of Esg-dependent suppression of Dof and FGF signaling.

Taken together, Esg acts as a central coordinator for tip cell specification in the fusion branch, first by modulating FGF signaling and second by controlling the FC-specific cell shape conversion (Kato et al., 2016). The robust maintenance of *esg* expression throughout fusion branch migration and fusion is central to the stereotyped branching pattern of the *Drosophila* trachea. An Esg-like tip cell regulator has not been identified in vertebrate blood vessels, which might explain the instability of tip cell fate in these blood vessels. The lack of a robust tip maintenance program might allow for the frequent conversion of tip cells and SCs that is essential for the flexibility seen in vessel remodeling.

## MATERIALS AND METHODS

### Fly stocks

The following fly stocks were used: *btl-Gal4* (Shiga et al., 1996), *UAS-GFP-moe* (Chihara et al., 2003), *HS-eGFP*, *HS-bnl* (Miao and Hayashi, 2015), *UAS-GFP-N-lacZ* (Shiga et al., 1996), *esg^[G66B]^/CyO* (Whiteley et al., 1992), *dys^2^*, *dys^3^* (Jiang and Crews, 2006) and *bs^[PZ]^* (Montagne et al., 1996). *UAS-esg* (Fuse et al., 1994), *UAS-btl wt* (Lee et al., 1996), *UAS-btl RNAi* (y[1] sc[*] v[1]; P{y[+t7.7] v[+t1.8]=TRiP.HMS02038}attP2), *UAS-dof/CyO* (Vincent et al., 1998), *btl-RFP-moe* (a gift from Markus Affolter) and *GRM13C07-Gal4* (*dys-Gal4*) (Jenett et al., 2012) were described previously and were obtained from the authors or from BDSC. The *esg_FC-Gal4* driver containing the FC enhancer of the *esg* genomic region was described in Kato et al. (2016). These stocks were cultured at 25°C.

### IR-LEGO

The IR-LEGO system (IR-LEGO-1000, Sigma-Koki Co., Ltd., Saitama, Japan) was combined with a confocal microscope (FV1000, Olympus) equipped with GaAsP detectors. The infrared (IR) laser was introduced through the lateral camera port of an inverted microscope (IX81, Olympus). The setting was as previously described (Miao and Hayashi, 2015).

### Live imaging

Confocal images were acquired using a laser-scanning confocal microscope (FV1000, Olympus) equipped with a PlanApo 60× NA 1.40 oil-immersion IR lens; 512×512 pixel images of 1 µm thick sections were captured every 1, 2, 3 or 5 min for 2 or 3 h with a 1×, 2× or 3× zoom with GaAsP detectors. The images were denoised and projected using the in-house software Malma (Kagayaki Kato, National Institutes of Natural Sciences, Okazaki, Japan).

### Immunofluorescence and antibodies

Primary antibodies were: mouse anti-dpERK (1:1000; Sigma-Aldrich, M9692, clone MAPK-YT); mouse anti-DSRF (1:1000; a gift from Michael Gillman); rat anti-Esg (1:100) (Fuse et al., 1994); rabbit anti-Dys (1:800; a gift from Lan Jiang); rabbit anti-Dof (1:200; a gift from Maria Leptin); and rabbit anti-β-gal (1:1000; Cappel). The chitin-binding probe (CBP) (1:50) was prepared from a bacterial expression construct according to a protocol provided by Yinhua Zhang (New England Biolabs). Secondary antibodies were: anti-mouse DIG biotin-sp-conjugated (used to enhance dpERK staining; 1:500; Jackson Laboratory, cat # 200-062-156); detected with HRP-conjugated Streptavidin (1:500; Thermo Fisher, N100) and TSA-direct Cy3 (1:50; Perkin Elmer, NEL704A001KT); anti-mouse IgG Alexa 546 (1:500; Molecular Probes, A-11003), anti-mouse IgG Alexa 633 (1:500; Molecular Probes, A-21050), anti-rat IgG Cy3 (1:500; Jackson Laboratory, 112-165-143), anti-rabbit IgG Alexa 555 (1:500; Molecular Probes, A-21429) and anti-rabbit IgG Alexa 633 (1:500; Molecular Probes, A-21071).
